# Urine Cell-Free DNA Integrity Analysis for Early Detection of Prostate Cancer Patients

**DOI:** 10.1155/2015/574120

**Published:** 2015-08-27

**Authors:** Samanta Salvi, Giorgia Gurioli, Filippo Martignano, Flavia Foca, Roberta Gunelli, Giacomo Cicchetti, Ugo De Giorgi, Wainer Zoli, Daniele Calistri, Valentina Casadio

**Affiliations:** ^1^Biosciences Laboratory, Istituto Scientifico Romagnolo per lo Studio e la Cura dei Tumori (IRST), IRCCS, Via P. Maroncelli 40, 47014 Meldola, Italy; ^2^Unit of Biostatistics and Clinical Trials, Istituto Scientifico Romagnolo per lo Studio e la Cura dei Tumori (IRST), IRCCS, 47014 Meldola, Italy; ^3^Department of Urology, Morgagni Pierantoni Hospital, 47121 Forlì, Italy; ^4^Department of Urology, Bufalini Hospital, 47521 Cesena, Italy; ^5^Department of Medical Oncology, Istituto Scientifico Romagnolo per lo Studio e la Cura dei Tumori (IRST), IRCCS, 47014 Meldola, Italy

## Abstract

*Introduction*. The detection of tumor-specific markers in urine has paved the way for new early noninvasive diagnostic approaches for prostate cancer. We evaluated the DNA integrity in urine supernatant to verify its capacity to discriminate between prostate cancer and benign diseases of the urogenital tract.* Patients and Methods*. A total of 131 individuals were enrolled: 67 prostate cancer patients and 64 patients with benign diseases of the urogenital tract (control group). Prostate-specific antigen (PSA) levels were determined. Urine cell-free (UCF) DNA was isolated and sequences longer than 250 bp corresponding to 3 genes (*c-MYC*,* HER2*, and* AR*) were quantified by Real-Time PCR to assess UCF-DNA integrity.* Results*. UCF-DNA was quantifiable in all samples, while UCF-DNA integrity was evaluable in all but 16 samples. Receiver operating characteristic analysis showed an area under the curve of 0.5048 for UCF-DNA integrity and 0.8423 for PSA. Sensitivity was 0.58 and 0.95 for UCF-DNA integrity and PSA, respectively. Specificity was 0.44 and 0.69, respectively.* Conclusions*. UCF-DNA integrity showed lower accuracy than PSA and would not seem to be a reliable marker for early prostate cancer diagnosis. Despite this, we believe that UCF-DNA could represent a source of other biomarkers and could detect gene alterations.

## 1. Introduction

Prostate cancer represents the most common tumor in men in Europe and the second leading cause of deaths from cancer in men [1]. Because of its slow development and potential curability in the initial hormone-dependent phase, prostate cancer is suitable for early diagnosis approaches [2]. Early diagnosis plays an important role in increasing disease-free survival and reducing mortality in patients with various tumor types, and noninvasive diagnostic procedures are more acceptable and have a higher compliance than invasive screening programs. Prostate cancer screening programs are based on the determination of prostate-specific antigen (PSA) in the blood. The use of this marker, which has been shown to reduce mortality, has recently become controversial [3–5]. PSA is not a tumor-specific marker as it can be synthesized by all prostate cells; elevated serum PSA levels can be found not only in prostate cancer patients but also in individuals with benign diseases of the urogenital tract such as prostatic hyperplasia, infection, and inflammation. Thus, the main limitation of this marker is the risk of false positives leading to overdiagnosis and -treatment in patients, with consequently higher healthcare costs [2, 5, 6].

A number of PSA derivatives have been proposed to improve the specificity of total PSA, including the percentage of PSA circulating in its unbound form (free PSA) which helps to distinguish between benign conditions and prostate cancer. Free PSA comprises several different isoforms including pro-PSA. Recently, the US Food and Drug Administration (FDA) approved the Beckman Coulter Prostate Health Index (phi), an approach that combines total PSA, free PSA, and pro-PSA. It has been demonstrated that phi is capable of increasing the specificity of total PSA alone, thus reducing the need for biopsy [7].

Although great efforts have been made to find new markers to complement or replace PSA, none has been fully validated or accepted. The only novel marker approved by FDA is PCA3, a messenger RNA detectable in urine samples from prostate cancer patients [8–10]. PCA3 in association with PSA has been proposed as a second level approach [9] as it appears to improve the performance characteristics of PSA [11]. However, despite showing high specificity, its coinciding low sensitivity has raised some doubts about its clinical value [11]. New diagnostic approaches that are robust, accurate, easy, and rapid to perform, with a favorable cost/benefit ratio, are thus still needed for prostate cancer.

Urine represents an important and promising source of biomarkers for cancer as urine-based diagnostic approaches are noninvasive and urine is readily available [12, 13].

Cell-free nucleic acids are known to be important for cancer diagnosis and prognosis [14], and the study of specific characteristics of urine cell-free DNA is ongoing to find biomarkers of urogenital tumors, including prostate cancer [15–18].

We previously focused our attention on urine cell-free DNA integrity in bladder and prostate cancers starting from the hypothesis that DNA from normal cells undergoing apoptosis is highly fragmented, whereas DNA from necrotic cancer cells maintains its integrity [15, 19, 20]. We performed a preliminary case-control study on prostate cancer, obtaining a sensitivity of 0.79 and a specificity of 0.84 in healthy individuals [16]. Starting from these promising results, we decided to enlarge the case series to include patients with prostate cancer or benign diseases of the urogenital tract, with the aim of validating our preliminary findings in this clinical setting in which patients appear to benefit more from an early diagnostic approach. We thus analyzed urine cell-free DNA fragments longer than 250 bp of three genes frequently amplified in prostate cancer:* c-MYC* (8q24.21),* HER2* (17q12.1), and* AR* (Xq12) [21–23].

## 2. Materials and Methods

### 2.1. Patients

The study was conducted on 131 individuals, 67 with a first diagnosis of prostate cancer and 64 with benign diseases of the urogenital tract (controls). The latter were divided into 3 main disease categories: prostatic benign diseases which include prostatitis, inflammation, prostatic benign hyperplasia, and adenomas (*n* = 26); kidney and bladder benign diseases consisting of kidney or bladder stones, cysts, and lithiasis (*n* = 24); and other diseases, including disorders of the testes, circumcision-related problems, and testicular hematoma (*n* = 14). Individuals with previous cancers were excluded from the study. Participants were enrolled from the Departments of Urology of Morgagni Pierantoni Hospital (Forlì, Italy) and Bufalini Hospital (Cesena, Italy). All provided written informed consent to take part in the study, which was reviewed and approved by IRST-AVR Ethics Committee. All prostate cancer patients underwent radical prostatectomy. The Gleason score and pathological stage were evaluated after surgical removal of the tumor. PSA levels were available in 110 patients. Median age was 68 years for prostate cancer patients and 62 for patients with benign urogenital diseases.

### 2.2. Urine Collection

First-morning voided urine samples were collected for UCF-DNA analysis. Specimens were collected from all patients before any surgical intervention. All individuals were instructed to give clean-catch urine samples, which were maintained at 4°C for a maximum of 3 h. Thirty-milliliter aliquots of urine were centrifuged at 850 g for 10 min and the supernatants were transferred into cryovials and immediately stored at −80°C until use.

### 2.3. UCF-DNA Analysis

DNA was extracted and purified from 2 mL of supernatant by Qiamp DNA minikit (Qiagen, Milan, Italy) according to the manufacturer's instructions. At the same time, DNA was extracted from a human prostate cancer cell line (LNCap) using the same kit. DNA was quantified by spectrophotometry (NanoDrop ND-1000, Celbio, Milan, Italy).

Real-Time PCR reactions were carried out by Rotor-Gene 6000 detection system (Corbett Research, St. Neots, UK) using IQ SYBR green (Biorad, Milan, Italy). Sequences longer than 250 bp corresponding to 3 oncogenes were analyzed:* c-MYC* (amplification product = 264 bp),* HER2* (amplification product 295 bp), and* AR* (amplification product 265 bp). A short 125 bp fragment of* STOX1* (locus 10q21.3), located in a region that is neither amplified nor deleted in prostatic tumors, was analyzed to check for potential PCR inhibition. Primer sequences were as follows:* c-MYC* fw 5′-TGGAGTAGGGACCGCATATC-3′, rev 5′-ACCCAACACCACGTCCTAAC-3′;* HER2* fw 5′-CCAGGGTGTTCCTCAGTTGT-3′, rev 5′-TCAGTATGGCCTCACCCTTC-3′;* AR* fw 5′-AGCCCAGGTTCTCTCCTGAT-3′, rev 5′-TGGCTAGTCCTCAGCTT-3′;* STOX1* fw 5′-GAAAACAGGGCAGCAAGAAG-3′, rev 5′-CAGACAGCATGGAGGTGAGA-3′. PCR conditions for the oncogenes were as follows: 95°C for 3 min followed by 45 cycles at 94°C for 40 s, 56°C for 40 s, and 72°C for one min. PCR conditions for the short* STOX1* sequence were as follows: 95°C for 90 s followed by 45 cycles at 94°C for 40 s and 54°C for one min. All Real-Time PCR reactions were performed in duplicate on 10 ng of each UCF-DNA sample. Various amounts of DNA from the LNCap cell line (0.01, 0.1, 1, 5, 10, and 20 ng) were also analyzed to construct a standard curve. The UCF-DNA value for each sample was obtained by Rotor-Gene 6000 detection system software using standard curve interpolation and the analysis was repeated if the difference between duplicate samples was greater than one cycle threshold. Standard curves were required to have an *R* ^2^ ≥ 0.98 to proceed with sample evaluation. The final UCF-DNA integrity value was obtained by summing the three-oncogene values. Real-Time experiments were performed independently in duplicate on the same 8 samples to test assay variability. The coefficients of variation (CV) were then calculated for* c-MYC*,* HER2*,* AR*, and* STOX1*. Real-Time PCR analyses were performed in accordance with MIQE guidelines [24].

### 2.4. Statistical Analysis

The relationship between the UCF-DNA integrity concentrations in the two subgroups was analyzed using a nonparametric ranking statistic test. The most discriminating cut-off values between patients with benign diseases and those with cancer were identified using receiver operating characteristic (ROC) analysis. True positive rates (sensitivity) were plotted against false positive rates (1 − specificity) for all classification points. Accuracy was measured by the area under the ROC curve (AUC), which represents an average probability of correctly classifying a case chosen at random. Study endpoints were sensitivity (the proportion of cancer patients who were correctly identified by the test or procedures) and specificity (the proportion of healthy individuals who were correctly identified), with their 95% confidence intervals (CIs). *P* values < 0.05 were considered statistically significant. Statistical analyses were carried out with STATA/MP 10.1 for Windows (StataCorp LP).

## 3. Results

Total free DNA concentration was quantifiable by spectrophotometry for all 131 samples, showing a median value of 3.5 ng/*μ*L (range 1.51–25 ng/*μ*L). A short fragment (*STOX1*, 125 bp) was analyzed to exclude the presence of PCR inhibitors. 115 samples showing an amplification of* STOX1* were then analyzed for UCF-DNA integrity, while the remaining 16 samples (10 from prostate cancer patients and 6 from patients with benign urogenital diseases) showed no amplification (not evaluable) and were excluded from the experimental and statistical analyses. Coefficients of variation (CVs) were calculated, considering 2 measurements of each gene in a series of 8 samples, to test the interim imprecision of each assay. CVs were <0.2 for all genes. PSA results were available for 110/115 patients. A full description of the case series is reported in Table 1.

No significant correlation was found between UCF-DNA concentration and UCF-DNA integrity (Spearman rank correlation test), suggesting that they are independent variables (data not shown). We did not observe a statistically significant difference in UCF-DNA concentration between prostate cancer patients and those with benign prostatic disease (Wilcoxon test *P* = 0.30).

**Figure 1:**
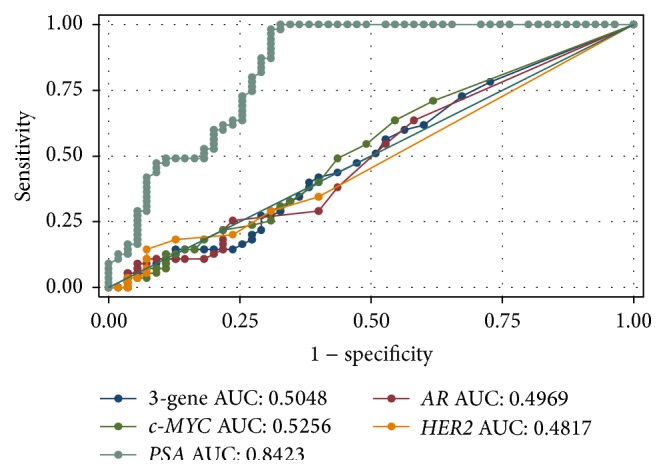
ROC curves of the sum of the three-gene concentrations (blue), c-*MYC* (dark green), PSA (light green),* AR* (red), and* HER2* (orange).

ROC curve analysis showed an AUC of 0.5048 (95% CI: 0.3963–0.6133) for UCF-DNA integrity and 0.8423 (95% CI: 0.7658–0.9188) for PSA (Figure 1). We also performed ROC curve analyses for individual genes: AUCs were 0.5256 for* c-MYC*, 0.4817 for* HER2,* and 0.4969 for* AR* (Figure 1). The AUC value for PSA was statistically higher than that of all the other markers. There were no statistical differences among AUCs of UCF-DNA integrity and those of individual genes.

Considering different cut-off values for UCF-DNA integrity analysis, sensitivity varied from 0.51 to 0.62, while specificity ranged from 0.40 to 0.49 (Table 2). The best cut-off value for UCF-DNA integrity was 0.04 ng/*μ*L.

The analysis of UCF-DNA integrity as a function of tumor characteristics did not highlight any significant difference between cancer patients with a Gleason score of ≤6 and those with a score of >6 or between pT2 and pT3 patients (Wilcoxon test *P* = 0.71 and 0.34).

The currently used standard PSA cut-off value (4 ng/mL) showed a sensitivity of 0.95 and a specificity of 0.69 in the overall case series, higher than that observed for UCF-DNA integrity at a cut-off value of 0.04 ng/*μ*L (Table 3). Specificity values of PSA and UCF-DNA integrity for the three categories of control patients were as follows: 0.39 and 0.43 for prostatic benign diseases, 0.91 and 0.43 for kidney and bladder benign diseases, and 0.91 and 0.64 for other diseases (Table 3).

## 4. Discussion

We performed UCF-DNA integrity analysis with the aim of verifying its role as an early diagnostic marker for prostate cancer, considering patients with benign urogenital diseases as controls. Our test exhibited a sensitivity of 58% and a specificity of 46% with an AUC of 0.5048 which did not confirm the findings of our preliminary pilot study [16], and the accuracy of UCF-DNA integrity was too low to be acceptable as an early diagnostic marker. Furthermore, in the present study PSA showed a better diagnostic performance in terms of sensitivity and specificity (95% and 69%, resp.) than UCF-DNA integrity. However, considering different categories of controls, we observed that PSA and UCF-DNA integrity had the same low specificity in benign prostatic diseases (39%), confirming that the PSA test has a high number of false positive results [3], especially when benign conditions such as prostatitis or adenomas are present.

We asked ourselves why these results were so different from those obtained in our previous case-control pilot study and came up with a number of possible explanations. First, the pilot study was composed of a small number of patients. Secondly, patients with inflammation, calculi, and cysts were enrolled in the present confirmatory study: these clinical conditions lead to the exfoliation of inflammatory cells in the urine which may increase the amount of cell-free DNA released, thus leading to false positive results. We also chose to focus on patients referred to a urology department for urological symptoms as we thought that this group would benefit most from early diagnosis in the event of cancer. We ruled out the possibility that DNA fragments passing through the glomerular filtration barrier influenced our analysis because such fragments are too short, as shown by Su and coworkers [25]. However, we cannot exclude that inflammatory cells exfoliated in the urine released long DNA, influencing our results. The low stability of urinary markers, dependent on urine pH and composition and on sample processing time, may also have affected our results. In addition, as epithelial cells of prostate origin make up only 10–20% of cells contained in urine samples [12], other cell types including inflammatory and bladder cells may have impacted the data obtained on UCF-DNA integrity. An important aspect of our work is that we chose to collect voided urine samples from patients before they underwent digital rectal examination (DRE); the number of tumor cells exfoliated in urine may consequently have been lower, reducing the sensitivity of the assay.

Despite its limitations and negative results, the present study highlighted the potential usefulness of urine cell-free DNA as a new source for prostate cancer biomarkers. As far as we know, there are no data in the literature on the use of cell-free DNA from urine supernatant to characterize prostate cancer. The few studies published on urine cell-free DNA alterations focus on their use as markers of early bladder cancer diagnosis [15, 17, 18].

We chose to analyze UCF-DNA integrity as it is a simple, cost-effective method with easily interpretable results. Although our data on UCF-DNA integrity are not encouraging because of the low accuracy observed, urine cell-free DNA was quantifiable in all samples and could thus prove to be an important source of biomarkers for prostate cancer. Other characteristics and alterations, such as epigenetic modifications, may be more accurate in distinguishing cancer from other benign diseases of the urogenital tract [26].

## 5. Conclusions

In the present study, although UCF-DNA integrity was not sufficiently accurate as a diagnostic marker of early prostate cancer, our results indicate the potential usefulness of UCF-DNA for prostate cancer characterization. Further research on other characteristics and alterations is needed to find accurate diagnostic, prognostic, and predictive markers.

## Figures and Tables

**Table 1:** Case series.

	Number of cases
Total number of patients	110^*∗*^

Prostate cancer patients	55
Gleason score	
6	19
7	25
8–10	8
Unknown	3
Pathological stage	
pT2a	13
pT2b	1
pT2c	13
pT3a	16
pT3b	1
Unknown	11
Median PSA (range)	6.32 (3.1–118.4)
Median UCF-DNA integrity (range)	0.06 (0–2.56)

Patients with benign diseases	55
Benign prostatic disease	23
Benign kidney and bladder diseases	21
Others	11
Median PSA (range)	1.38 (0–14.00)
Median UCF-DNA integrity (range)	0.06 (0–3.41)

^*∗*^Patients without PSA and UCF-DNA integrity results were excluded; UCF: urine cell-free.

**Table 2:** Sensitivity and specificity of different UCF-DNA integrity cut-off values in the overall case series.

Cut-off (ng/*µ*L)	Sensitivity	Specificity
0.03		
*n*	34/55	22/55
Rate (95% CI)	0.62 (0.48–0.75)	0.40 (0.27–0.54)
0.04		
*n*	**32/55 **	**24/55 **
Rate (95% CI)	**0.58 (0.46–0.73)**	**0.44 (0.30–0.58)**
0.05		
*n*	31/55	26/55
Rate (95% CI)	0.56 (0.42–0.70)	0.47 (0.34–0.61)
0.06		
*n*	28/55	27/55
Rate (95% CI)	0.51 (0.37–0.65)	0.49 (0.35–0.63)

UCF: urine cell-free.

**Table 3:** Sensitivity and specificity of UCF-DNA integrity and PSA.

	Sensitivity	Specificity			
		Overall case series	Benign prostatic disease	Benign kidney and bladder disease	Others
PSA^*∗*^					
*n*	52/55	38/55	9/23	19/21	10/11
Rate	(0.95)	(0.69)	(0.39)	(0.91)	(0.91)
UCF-DNA integrity^*∗∗*^					
*n*	32/55	24/55	10/23	9/21	7/11
Rate	(0.58)	(0.44)	(0.43)	(0.43)	(0.64)

^*∗*^PSA cut-off value: 4 ng/mL.

^*∗∗*^UCF-DNA integrity cut-off value: 0.04 ng/*µ*L.
